# Six attachment discourses: convergence, divergence and relay

**DOI:** 10.1080/14616734.2021.1918448

**Published:** 2021-05-12

**Authors:** Robbie Duschinsky, Lianne Bakkum, Julia M. M. Mannes, Guy C. M. Skinner, Melody Turner, Alissa Mann, Barry Coughlan, Sophie Reijman, Sarah Foster, Helen Beckwith

**Affiliations:** a Department of Public Health and Primary Care, University of Cambridge, Cambridge, UK; b Department of Clinical Child and Family Studies, Vrije Universiteit Amsterdam, Amsterdam, The Netherlands; c Department of Psychology, University of Bath, Bath, UK; d Institute of Criminology, University of Cambridge, Cambridge, UK; e Center for Early Intervention and Family Studies, University of Copenhagen, Copenhagen, Denmark; f Department of Social Work, Education and Community Wellbeing, Northumbria University, Newcastle upon Tyne, UK

**Keywords:** Attachment, child welfare, developmental science, psychotherapy, sociology of science

## Abstract

Attachment concepts are used in diverging ways, which has caused confusion in communication among researchers, among practitioners, and between researchers and practitioners, and hinders their potential for collaboration. In this essay we explore how attachment concepts may vary in meaning across six different domains: popular discourses, developmental science, social psychological science, psychiatric diagnosis, psychotherapy, and child welfare practice. We attempt to typify these forms of attachment discourse by highlighting points of convergence, divergence, and relay between the different domains. Our general conclusions are that diversity in the use of attachment concepts across different domains of application has been largely unrecognised, and that recognition of these differences would reduce confusion, help identify sites where infrastructure needs to be developed to support coordination, and strengthen opportunities for collaboration to mutual benefit. We suggest that academic attachment discourse would benefit from clarification of core terminology, including: “attachment”, “internal working model”, “trauma”, and “dysregulation”.

## Introduction

The occasion for this target article and special issue is the publication of *Cornerstones of Attachment Research* (Duschinsky, 2020) by Oxford University Press. The book is available for free download thanks to open access funds from the Wellcome Trust. *Cornerstones* analyses the work of five research groups: Bowlby and his collaborators; Ainsworth and her students; Main, Hesse and the Berkeley Social Development project; Sroufe, Egeland and the Minnesota Longitudinal Study of Risk and Adaptation; and the work of Shaver and Mikulincer and their collaborators. Each chapter draws from a comprehensive study of published work by the researchers, how their results and ideas were received by others, as well as access to additional sources, such as unpublished books by Bowlby and Main. Alongside composition of *Cornerstones*, where possible we have brought important unpublished sources into print (e.g. Duschinsky & White, 2020).

Reflecting on developmental science, Ainsworth (1972, p. 126) observed that “in terms of his problem, theoretical orientation, resources, opportunities, and personal style, each investigator chooses his own set of compromises. The interests of science seem likely to be best served in this context by a multiplicity of studies, each with its own compromises”. In line with this, the focus in *Cornerstones* on five research groups was intended to provide a basis to compare and understand the particular challenges and contributions of some key researchers, as a lens on the wider predicament of attachment science. Yet *Cornerstones* was by no means an attempt to comprehensively characterise this area of research as a whole. It is one contribution to a wider historical literature (e.g. Van der Horst, 2011; Van Dijken, 1998), and there is much that it was not able to cover. Additional work is on its way, including *Mentalisation and Epistemic Trust* (Duschinsky & Foster, 2020), a book addressing the research and ideas of Fonagy and colleagues at the Anna Freud Centre, which will also be free to download.

In this essay we use historical and sociological analysis to draw out one of the themes of *Cornerstones*: the diverging ways in which attachment concepts are used across scientific, applied, and popular domains, often without recognition of this divergence. There is no problem in divergence, and to an extent it is inevitable. The problem, we propose, is that the divergence is not recognised and dealt with, which may lead to confusion in communication among researchers, practitioners, and the general public. Bosmans (2016) has observed that there is comparatively good coherence in the use of concepts between researchers and clinicians in the tradition of cognitive behavioural therapy, and infrastructure to support this coordination. This is in contrast to attachment concepts, which, as we illustrate over the course of the next sections, are more likely to be overfull of contradictory meanings, allowing individuals and groups to talk past one another and hindering their potential for collaboration.

To respond to this problem, our intended contribution here has been the attempt to typify these divergent forms of attachment discourse. In doing so we have sought to acknowledge that each is shaped by distinct social contexts, the priorities of these contexts, and their criteria for what counts as relevant and dependable knowledge and measurement, as well as criteria for who is considered a credible speaker. In terms of the problem we are seeking to address, an analogy from statistics would be the issue of measurement invariance, which asks whether a construct is measuring the same thing across different groups. Whereas *Cornerstones*, as a book, has space to be detail-focused, this essay is necessarily more schematic; readers may consult *Cornerstones* for the full arguments and referencing supporting the claims made here. Our ambition here is to capitalise on the advantages of schematism, sketching some critical lines of difference in the use of attachment concepts by considering four questions:

(1) What is the context of the account of attachment?
(2) What do accounts of attachment demand from their concepts, given their contexts?
(3) How do accounts of attachment handle the mismatch between the contextual demands on them, and the properties of actually available attachment theory and research?
(4) What are the points of relay – of dependence and articulation – between different accounts of attachment?

Our inquiry here runs against the assumption, appearing among proponents but especially among critics of attachment theory, that the strengths and weaknesses of the paradigm are the same across its different contexts of application. We apply the four questions above to consider points of convergence, divergence and relay between six domains: popular discourses, developmental science, social psychological science, psychiatric diagnosis, psychotherapy, and child welfare practice. We do not intend to suggest that individuals and groups are forced in any simple way to follow dominant discourses, or lack awareness of historical and sociological processes. In fact attachment researchers have sustained reflexive commentary over decades on the state and challenges of attachment research, and drawn on these reflections to inform their studies (classic works include Van IJzendoorn & Tavecchio, 1987; Waters & Sroufe, 1983).

Furthermore, our intention is to highlight, and by no means to negate, the fact that there are important links between the different domains. There are many nodal individuals and groups between these networks of discourse and practice: for instance Charles Zeanah and colleagues bridge developmental and psychiatric discourses; and Peter Fonagy and Arietta Slade and their colleagues bridge developmental and psychotherapeutic discourses. There are also structural nodes between discourses, such as the “implications” sections of papers, in which academic attachment discourse can transition into one of the other forms. We hope that our typifications of a heterogeneous and contested reality may offer constructive insight into certain tendencies in the contemporary situation of attachment theory and research, whilst also providing a frame for considering practices that differ from our typification, some of which are discussed in *Cornerstones*.

### Popular attachment discourse

#### Context

During the 1950s, Bowlby addressed various audiences: academic, clinical and wider publics. Bowlby’s popular writings in this period represented less developed and qualified ideas than in his later scholarly work; these ideas were also further simplified and yoked to existing popular assumptions and metaphors to increase their accessibility. This helped the ideas travel into public discourse, which would not have been as easy if accompanied by technical depth and qualifications. Today, there are a variety of ways in which attachment is discussed in the media. Some are accurate whilst retaining accessibility; a prime example are those by Sheri Madigan (https://www.madiganlab.com/additional-resources). However, the legacy of Bowlby’s early popular writing, together with the inaccessibility of academic attachment discourse (e.g. behind paywalls; little public engagement from 1970–2000s) have helped give rise to a potent and pervasive popular attachment discourse. Popular attachment discourse is nested in and circulates through mass market books, magazines, internet forums, new social media, policy reports, and other public forms in which a “quick hit” is expected, and depth penalised. The family and parenting are perennial objects of public concern; in part this is because the family is the site of reproduction of the public. Popular discourses claim the authority of “public outrage” to make judgements about normal and abnormal forms of parenting. Attachment is invoked to speak to societal concerns about parenting and the family, and intersects with gendered assumptions about these institutions, often in mutually reinforcing and moralised ways.

#### Desires

Popular attachment discourse must walk a tightrope: it needs to retain resonance with ordinary language and its assumptions in order to be appealing, presenting a quality of obviousness and intuitiveness, whilst also appearing to offer something extra in terms of authority, depth or insight. It must be “light enough to travel”: able to plug into various ordinary situations and feel relevant. Over the past decade, the scientific appearance of popular attachment discourse has been reinforced through appeal to neuroscience, which makes claims appear more real and founded.

#### Adjustments

To make attachment theory and research meet the demands of the popular context, popular attachment discourse has some distinct qualities. At a theoretical level, Bowlby’s behavioural systems model of the mind is cut. Citations are generally to the early Bowlby, or to neuroscientific glosses on Bowlby’s emphasis on the importance of early care. Meta-analytic research is ignored wholesale in popular attachment discourse, since this would require much more moderate and tempered claims. Attachment is often treated as a synonym of close relationships and used not only to describe a child’s bond to a key caregiver, but also a parent’s “attachment” to their child. Individual differences in attachment have generally been formatted to focus on the divisions between “good” and “bad”, and “strong” and “weak” attachments. Though popular attachment discourses with stronger links to developmental or social psychological attachment discourses tend to divide between “secure” and “insecure”. Nonetheless, where security and insecurity are invoked, popular attachment discourse has an affinity for treating the Ainsworth categories as natural kinds, and disorganised attachment as chaotic, a state of “madness”. In part, this is because “how the sausages are made” in coding the Strange Situation and other aspects of methodology are passed over, as well as the relative applicability of Ainsworth’s classifications after infancy; this makes the categories seem more like natural kinds. In part, the Ainsworth classifications are interpreted through a folk psychology that assumes category membership is: fixed; non-overlapping; lacking in internal heterogeneity; rooted in hidden causal processes which achieve particular functions; and that deviation from categories mean a thing is broken (Atran, 1998).

#### Relay

Popular attachment discourse has drawn from the academic authority of developmental attachment research, as well as the concept of “attachment disorders” from psychiatric discourse. Attempts to qualify and circumscribe the object of these discourses have not entirely hindered this authority, since technical debates struggle to circulate beyond their immediate sphere. Popular attachment discourse has also been fed by the role of appeals to attachment within child welfare, which make attachment seem relevant to ultimate judgements about acceptable and unacceptable parenting, and the responsibility of the public to address this (e.g. Building Greater Britons, 2015). Social psychology has also entered popular discourse concerned with the formation and maintenance of well-functioning couples, thanks especially to the idea, implied by Hazan and Shaver’s early “love quiz” measure, that “attachment styles” are transparently available to individuals themselves. The contemporary idea in social psychology of attachment anxiety and avoidance as latent factors, with meaning beyond an individual’s self-knowledge, has not had the same appeal for popular attachment discourse. The Adult Attachment Interview (AAI) has likewise has not been picked up by much popular attachment discourse.

### Attachment research in developmental science

#### Context

Attachment emerged as a thriving paradigm within developmental psychology in the 1970s, led initially by Mary Ainsworth’s research group, but with important groups subsequently emerging in Berkeley, Minnesota, Regensburg and SUNY in the 1980s, and Pennsylvania, Harvard, Leiden, Maryland, Haifa and London by the early 1990s. This domain of attachment discourse has been firmly nested within the wider terrain of academic developmental science, and forms a standard part of the curriculum taught to students. The filiation of developmental attachment research from Ainsworth has functioned as a form of symbolic authority, one that social psychologists have publicly criticised in the 2000s as an obstacle to the acceptance of work by social psychologists (e.g. Shaver & Mikulincer, 2002; see also Spies & Duschinsky, 2021). The laboratory setting favoured by developmental science has allowed the control of variables and close observation of subtle behaviours, helping sustain the authority of this discourse as a form of scientific knowledge. Where this might be put at risk, researchers have tended not to go, for instance, study of reunions in daycare settings or following therapy would have been a much more scalable assessment than the Strange Situation, and contributed to lower costs, larger sample-sizes, and better links to professional practice. Yet few attachment researchers have examined reunions with parents in daycare settings or following therapy (exceptions include Bick et al., 2012; Steele et al., 2019). Developmental science has been especially focused on the developmental foundations of social competence and mental health, perhaps due to the legacy of Bowlby’s theory or their clinical relevance, but perhaps also as a result of their relevance to potential funders. In sociological perspective, social competence and mental health can be seen to correspond to an individual’s external and internal functionality.

#### Desires

Developmental attachment discourse can be typified as oriented by four interrelated desires. First, the paradigm has sought evidence of external validity, through attempts to demonstrate a nomological network of predictable correlates, especially in the domains of mental health and social competence. Second, developmentalists have sought convergent validity as functional legitimacy for their constructs. This has had special priority, given small samples and high barriers to effective use of complex observational measures such as the Strange Situation and AAI. Third, developmental attachment discourse has explored a vast array of correlates, to varying degrees driven by theory (Waters et al., 2005). Yet, within this diversity, a particular focus has been attempts to understand developmental processes relevant to the basis of individual self-regulation or dysregulation. Fourth, developmental attachment research has been oriented by the desire to have work accepted by the wider field of developmental psychology (e.g. journals, grants), providing the economic and symbolic capital necessary for the reproduction of the paradigm within academic life.

#### Adjustments

Developmental attachment discourse has made several adjustments to meet these desires. Forms of attachment have been formatted to focus on the divisions secure/ insecure and organised/disorganised. Subtypes appear to have no currency for the project of sustaining and furthering attachment research within generalizable science with adequate statistical power. For instance, though van IJzendoorn and colleagues (1983) speculated on the meaning of the B4 subclassification, this has not been tested when data later became available to do so. The ambivalent/resistant attachment has, for the same reason, been frequently amalgamated in analyses into an overarching “insecure” category, though there are exceptions. Another adjustment has been a doubling down on the Strange Situation and AAI to sustain convergent validity, and a focus on individual differences more than normative processes. In this focus on individual differences, further elaboration of the idea of behavioural systems has been neglected (e.g. the questions of whether anger is a behavioural system; whether a dominance system could shed light on controlling-punitive behaviour), in favour of a theoretical focus on “minimising” and “maximising” of attachment. And in the conceptualisation of “minimising” and “maximising”, as documented in Duschinsky (2020), the dominant frame of reference has been Cassidy’s (1994) interpretation of these in terms of regulation, social competence and mental health, and not Main’s (1995) model of attentional processes.

Besides these, we would emphasise two adjustments of particular importance for developmental attachment discourse. A first was the early adoption of meta-analysis, as a tool for attesting to credible, convergent knowledge and for understanding moderators of disappointment, for instance the “transmission gap” between the AAI and Strange Situation indexing the structural need to gain capital in developmental science by reporting large effect sizes (see e.g. the discussion in Verhage et al., 2016). A second adjustment was an implicit acceptance by the generation of developmental scientists after Ainsworth, that research using measures like the Strange Situation and the AAI could only be a small and technical area of academic work (see e.g. Waters, 1983, advocating that only trained coders should use the Strange Situation, and announcing the commencement of accredited training). The Strange Situation and AAI are penetrating, resource-intensive, and must generally be understood in depth by a researcher in order for their work with the measure to contribute meaningfully to theory in the developmental tradition. This combination has led to the construction of an oral culture of training institutes, unpublished coding manuals, and mentorship by established developmental attachment researchers for transmission of the tradition. This solution was not inevitable – priority could have been given to testing abbreviated versions of measures (e.g. Caron et al., 2018, using a cut-down AAI) decades earlier. Dependence on an oral culture risks certain in-group dynamics and assumes the inevitability of a small possible number of credible developmental attachment researchers. Given the intensified imperative for psychometric credibility, generalisability and scalability in contemporary developmental science, this solution has begun to see significant renegotiation (see Schuengel et al., this issue). In sociological perspective, perhaps the most important question is whether developmental attachment research is still professionally rewarding enough to recruit able early career researchers. So far, in our view, that has remained the case, though the unusual time-investment required for credibility as a developmental attachment researcher constricts recruitment, for instance, by making it difficult for able researchers in cognate areas to put a toe in the water.

#### Relay

Our studies have suggested that developmental attachment research has been rather sealed off from popular and psychiatric attachment discourses, oriented much more by the internal demands of academic life. There has, however, been significant relay from therapeutic discourses. For instance, the concept of “mentalisation” has served as a “boundary object”, incorporating sufficiently diverse meanings as to suture – and at times mask – gaps between the concerns of therapists and researchers (as acknowledged by Fonagy & Allison, 2012). Likewise, theorising about disorganised attachment by researchers has been influenced by the connotations in therapeutic discourse of the idea of “disorganisation” (Reijman et al., 2018). An important development in the past decade for developmental attachment research has been the increasing relay from social psychology, where standards for psychometric credibility, generalisability and scalability rose faster in the 1990s and 2000s than developmental science. The strategies developed by social psychologists – such as replacement of attachment categories with dimensions constructed using taxometric methods and validation of short versions of existing measures – have gained increasing hearing among developmentalists. Chris Fraley and Glenn Roisman have been nodal figures in this shift.

### Attachment research in social psychology

#### Context

Social psychology attachment discourse is nested within the wider terrain of social psychology, and cognate areas of personality and experimental psychology. In the early 1980s, Shaver and colleagues used self-report methodology to explore individual differences in attachment. Initially, the Ainsworth Strange Situation categories were extrapolated into “attachment styles”, readily knowable and reportable by the individual themselves. However, Brennan et al. (1998) identified attachment anxiety and avoidance as two latent factors in individual differences in attachment, using these latent factors to create the Experiences in Close Relationships scale (ECR). Attachment anxiety and avoidance are now seen as latent processes that can shape perceptions, attitudes, and preferences, without the individual’s awareness. This has been especially reflected in research examining the effect of attachment primes on behaviour. Where such findings prove replicable, they offer particular credibility in promising to isolate causal processes.

#### Desires

Social psychological attachment discourse can be typified as oriented by four interrelated desires. First, the paradigm has sought evidence of external validity of the treatment of adult relationships as attachment relationships, through attempts to demonstrate a nomological network of predictable correlates, especially in the study of strategies for responding to potential stress in romantic relationships. Through these efforts, they have achieved recognition as a legitimate branch of attachment research, despite lacking the filiation from Ainsworth available to the developmentalists. Second, given low barriers to the creation of ever-more self-report measures, there has been a structural need for methodological convergence, epitomised by the creation of the ECR in the 1990s. Third, attachment research in social psychology has generally sought to shed light on individual social preferences and attitudes towards togetherness in relationships. This can be regarded as a specific form of the wider liberal frame of reference in social psychology, which registers humans at the point that they can act independently, though the concern is then with how they ultimately live together (Stainton Rogers et al., 1995). Fourth, acceptance has been sought from the wider field of social psychology (e.g. journals, grants), providing the economic and symbolic capital necessary for the reproduction of the paradigm within academic life.

#### Adjustments

Social psychological attachment researchers have negotiated the tradition of Bowlby and Ainsworth in new ways, shaped by their concerns. For instance, the idea of completely independent and distinct internal working models of “self” and of “others” was excavated by social psychologists from Bowlby (1973) even though the claim that models of “self” and “other” are independent only appears once in his writings. Elsewhere Bowlby used the term to indicate that we develop expectations about self-interacting-with-others, not independent and distinct individual attitudes towards “self” and “others”. To take another example: whereas developmentalists have left Bowlby’s notion of behavioural systems gathering dust on the shelf, Shaver, Mikulincer, and colleagues have given this idea extensive consideration, and developed scales for “anxious” and “avoidant” forms of other behavioural systems, mixing their labour with Bowlby’s legacy on topics outside those symbolically owned by the developmentalists. Social psychologists have also by and large limited their interest to adequately functioning individuals who are capable of exercising autonomy. Less concern has been given to samples where both high anxiety and high avoidance might be expected, producing conflict within attitudes and preferences. Little attention has also been paid to parenting: at best, self-report measures would draw with the AAI in predicting parenting, and there was the possibility that they would lose. This would risk hindering the standing of the social psychological tradition with developmentalists, for whom, since Ainsworth, the study of attachment overlaps extensively with the study of parenting.

Another adjustment has been the use of factor analysis to establish theory and methodological convergence. On the one hand, this approach has helped achieve credibility in the field of social psychology generally, and helped to sustain consensus. However it arguably shut the box too quickly and tightly on theoretical questions about the meaning of the “minimising” and “maximising” of attachment. For instance, items were smuggled aboard the ECR assessing passivity and aggression, which cloud associations between the ECR and passive or aggressive behaviour. More importantly, the presumption of two latent factors has led to widespread disinterest in growing evidence suggesting that security is its own factor, not reducible to the absence of anxiety or avoidance (a notable exception is Gillath et al., 2009). To give another example: whilst Shaver and Mikulincer’s thinking about a dominance behavioural system is potentially a fruitful development for attachment research, their account has been undermined by problems in conceptualising what the minimisation of dominance might mean.

#### Relay

Attachment research by social psychologists has drawn extensively on the terminology of the developmental tradition. However, as we document in *Cornerstones* and elsewhere, almost every such term is used with a different sense between the two traditions, contributing to extensive miscommunication. For instance by “internal working models”, social psychologists mean the elaborated symbolic and affective representations made by humans about attachment figures and their availability, and the value of the self to these attachment figures. Developmental scientists generally mean expectations about the availability of attachment figures. An exception are those developmentalists interfacing strongly with psychotherapy, who tend towards appeal to the ordinary language connotations of terms, which sometimes and incidentally tends to align them with social psychological uses of these terms.

### Attachment in guidance for psychotherapy

#### Context

Bowlby ([1985] 2020) recalled his development of attachment theory as an attempt to provide a model for psychotherapy, more than the basis for a paradigm for empirical research. In the mid-1990s, many texts were produced with an audience of psychotherapists and other psy-practitioners in mind, which drew in varying ways, and to varying degrees, on the authority and insights of the theory offered by the developmental tradition. Jeremy Holmes, Allan Schore, Patricia Crittenden, Daniel Hughes, Sue Johnson, and Peter Fonagy may be listed as among the many prominent advocates of attachment to psychotherapists. From the 2000s, this theory-based guidance has been elaborated, qualified, and contested by guidance based on empirical research with patients (Berry & Danquah, 2016; Steele & Steele, 2018). This domain of attachment discourse has been supported by the availability of attachment concepts for supporting clinical formulation by presenting claims that are encompassing, intuitive, and potentially insightful, and by the passion of contemporary clinical organisations (e.g. responding to the bureaucratic logic of the National Health Service; the demands of health insurers in the US) for categories as the basis of action and allocating resources. The interest of clinicians and clinical organisations has helped sustain a market for commercial trainings and associated books.

#### Desires

Attachment discourse in works for psychotherapists can be typified as oriented by four interrelated desires. First this discourse requires an evocative vocabulary about human relating, permitting wide perceptions of relevance by practitioners supporting clients with different problems; it must walk a similar “tightrope” to popular attachment discourse. Second, it must offer to make sense of psychological distress in its complexity, especially with respect to transdiagnostic factors, as well as suggest next steps. Third, it must appear to have the imprimatur of science, permitting “evidence-based practice”. Finally, there is a general desire for scales with cut-offs or categories for assessment of attachment, giving information for formulation and judging thresholds for interventions.

#### Adjustments

Perhaps the most important adaptation of attachment in guidance for psychotherapists, compared to academic uses of the same concepts, has been that insecurity is framed as the *mechanism* of all mental pathology rather than a correlate. For instance, Fonagy and colleagues (e.g. Fonagy & Allison, 2012) have situated non-mentalising as the basis for most mental health disorders, and as occurring when the attachment system is activated without access to the capacity to reflect on thoughts and feelings. Crittenden interprets individual differences in attachment as the basis of information-processing strategies responsive to an individual’s history and current environment, again treated as implicated in most forms of mental ill health (see e.g. Crittenden, 2017, on autism).

#### Relays

Guidance for therapeutic practice based on attachment theory has drawn primarily, if not exclusively, from the terminology – though not the operationalization – offered by developmental attachment research. Popular attachment theory and child welfare attachment discourse have also helped support a “surround sound” effect of mutually supportive discourses giving the impression that a thing called “attachment” is important for children’s wellbeing, though at the price of absorbing some moralisation about family life (e.g. parental gender norms), and at the price of significant mystification of what is actually meant by attachment.

### Attachment in psychiatric diagnosis

#### Context

Our empirical work has identified that attachment discourses have had various forms of influence on psychiatric diagnostic practices. For instance, as Coughlan has documented, they may appear in differential diagnosis of children showing socioemotional and communication difficulties, where alternatives might be ADHD and autism spectrum disorder (Coughlan et al., 2021). Our focus here will be on the entry of attachment to psychiatric diagnosis as the attachment disorder diagnosis, since this is the primary formal location of appeal to attachment. We have papers forthcoming on other uses of attachment within diagnostic practice.

In 1980, the “Infancy, Childhood and Adolescent Disorders” committee of the Statistical Manual of Mental Disorders (DSM-III; American Psychiatric Association, 1980) introduced the category of “reactive attachment disorder in infancy” as a recognised diagnosis. This was an attempt to bring within medical assessment practice observations that had been made of behaviours shown by institutionalised and former institutionalised children, drawing on Bowlby’s influential 1951 report to the World Health Organization, as well as other reports. Bowlby’s work after 1958 – i.e. his theory of attachment – was not incorporated into the conceptualisation. Initially, the disorder was to be diagnosed on the basis of weak infant physical growth, poor social responsiveness, and emotional apathy, as a consequence of grossly inadequate experiences of caregiving. In 1987, the diagnosis was revised to remove the physical growth criterion and adjust the age criterion, and has since seen other changes. Since the 1990s, there has been slowly growing research concern with attachment disorders. A lack of infrastructure to develop, specify and regulate use of the category has contributed to concerns about both over- and under-diagnosis of “attachment disorders”, as well as leaving space for quackery in the marketing of promised commercial cures (Allen & Schuengel, 2020). Taken together, attachment disorder as a psychiatric classification can be attributed to the intersection of two contexts. The first is the role of psychiatric nosology in the structuring of mental health services and billing. A second is institutional care and unstable foster care, which can allow children to grow up without a stable attachment figure.

#### Desires

Psychiatric discourse on attachment disorder, firstly, seems concerned to shed light on the way that some children, following chronic experiences of insufficient and/or highly unstable care, may fail to seek and respond to comfort from others, leading to disruptions in mental health and social competence. The category is a hinge between an ostensive cause (a history of insufficient care) and a set of behaviours (failure to seek and respond to comfort). The presumption is that the diagnosis allows the behaviours to be interpreted, making sense of clinical complexity. Though there is as yet little scientific evidence for treatment for attachment disorders. Researchers working with the attachment disorder category have sought to establish a scientific basis for it sufficient for its retention as a psychiatric diagnosis.

#### Adjustment

Comparing psychiatric attachment discourse to the other five forms of attachment discourse considered here, the lack of lexical crossover is striking: they seem to only have the word “attachment” in common. Viewed as a network, psychiatric attachment discourse is quite isolated from the others. Though there has been some work attempting to consider how the category relates to the tradition of developmental attachment research. This has been hindered by a lack of conceptual clarity about the meaning of “insufficient care”, and by inconsistency in assessment practices. In recent years, behaviours suggesting indiscriminate behaviour towards adults have been conceptualised as less integral to the attachment disorder construct than failure to seek care, which promises to tighten the relationship between theory and diagnosis (Zimmermann & Soares, 2019).

#### Relays

Psychiatric attachment discourse drew from Bowlby’s early work on institutionalisation, but then for decades ran independently of the developmental tradition of attachment research. This helped leave space for an intersection of popular attachment discourses with the underdeveloped psychiatric category, contributing to the emergence of spurious “attachment therapies” (e.g. holding therapy). In past decades some attempts have been made at rapprochement between psychiatric and developmental attachment discourses, especially as developmental researchers have acknowledged the need of adoptive and foster parents, and the clinicians working with them, to understand their children’s behaviour.

### Attachment as child welfare

#### Context

In the 1950s and 1960s, Bowlby was active in advocating the relevance of his ideas for child welfare practice. Attachment theory offers welfare professionals a framework that appears to predict later risk to a child’s health and development from the child or parents’ observable behaviour. Over the years, both social work academics and policy documents have encouraged welfare professionals to use the image of secure attachment as the point of comparison when making assessments of parenting capacity. Today, attachment as child welfare discourses are nested within the institutional framework of the children’s workforce, safeguarding practice, and the family courts. In a survey conducted by the UK Department for Education (2018) of organisations working with children in need of help and protection, attachment theory was, by a large margin, cited as the most frequently used underpinning perspective.

#### Desires

This domain of attachment discourse appeals to attachment concepts to signify childrens’ welfare and best interests, delivering categorical judgements about the basis for action in predicting and preventing future harm (Forslund et al., 2021). In this way, attachment is sought as a way to make sense of complexity and minimise uncertainty, which together promise to specify risk and therefore indicate next steps. This usage stands in conflict with the anti-labelling values of some of the children’s workforce; this reflects a broader conflict for helping professionals within contemporary risk-focused institutions.

#### Adjustments

Attachment as child welfare discourse tends to deploy attachment categories as if they were quasi-diagnostic. Whereas clinical diagnosis is a form of judgment restricted to clinical professionals, attachment categories are not regulated. There have been social work academics who have advocated for – and offered commercial trainings in – the use of the categories in child welfare practice, without the need for specialist training. Individual differences in attachment have generally been formatted to focus on the divisions between “good” and “bad”, and “strong” and “weak” attachments, though at times “insecure attachment”, “attachment disorder”, “attachment issues”, or “attachment problems” are invoked to signify a prognosis requiring state intervention. Nonetheless, these appeals to attachment may not be regarded as credible by the family courts (see e.g. G.M. v. Carmarthenshire County Council & Anor, 2018), and many practitioners are unsure how best to draw attachment theory into child welfare practice. This predicament can contribute to extremes of overzealous use and aversion to ideas of attachment, alongside some hesitant use (e.g. North, 2019). As with popular attachment discourse, parents may be described as “attached” to their child.

Some of this uncertainty may manifest from a lack of available, or indeed sufficiently specific and sensitive, measures for assessment of individual cases according to psychometric criteria. There is also uncertainty about how practitioners are meant to use attachment concepts without specialist training in placing children into attachment categories. Some practitioners therefore seek further training in assigning attachment categories (e.g. Dallos et al., 2020), while other researchers advocate for a move from static diagnoses to assessments of the potential for enhanced parenting (Van IJzendoorn et al., 2018). Though there is yet to be any evaluation of whether child welfare assessment informed by attachment is superior to assessment as usual, in part due to the weak networks linking child welfare practice and academic attachment research.

#### Relays

A marked quality of attachment as child welfare discourse is that, especially in its less sophisticated forms, concepts are drawn without particular distinction from popular, developmental, therapeutic and psychiatric discourses. However it may be noted that access to developmental attachment research or psychiatric research is very rarely direct, and generally mediated by texts written by popularisers who are not themselves trained in the relevant measures. This has at times led to inadvertent but profound mischaracterisation of the available research in works for welfare practitioners (e.g. Pearce, 2009).

## Conclusion

Bowlby wrote and spoke for a variety of different audiences, writing numerous magazine articles, academic works, and works for applied practitioners in psychiatry, psychotherapy and child welfare. However, Ainsworth and the generation following her withdrew from exogenous engagement, except to an extent with psychotherapists, contributing to the fragmentation of the meanings of attachment discourse across groups ostensibly using the same ideas. This was despite a proliferation of program and policy applications of attachment ideas during the period. Such disengagement has been shifting in recent years, as researchers have sought to be more responsive to the concerns of popular and applied attachment discourses. The shift coincides, in a macro perspective, with growing concerns with the democratic standing of academic research since the 1990s, and more locally with changes in the concerns of attachment research away from distinctions between attachment classifications, and towards intervention research (Schuengel et al., this issue). We anticipate that any “move to the level of collaboration” will be helped first by wider recognition of the various discourses as we have presented here, and second, by some conceptual spring cleaning. As a start, we propose a clarification of academic attachment discourse particularly in the following six areas:

(1) Where any fine work needs doing, academic discourse would benefit from avoiding shorthands where it is assumed that others will pick up the intended meaning. For instance rather than the overladen term “attachment” it may be preferable to specify e.g. “perceived safe haven availability”. Waters and Waters (e.g. 2006; Waters et al., 2020) have been arguing along related lines for some years, and Thompson et al. (2020) have recently made similar claims.
(2) The model of “minimising” and “maximising” of attachment needs further scrutiny. Main’s attentional theory should be translated into testable hypotheses, including whether security has attentional properties irreducible to the absence of “minimising” or “maximising” forms of insecurity.
(3) The term “expectations” could be used instead of “internal working model” when this is what is meant. Where something else is meant, this should be spelt out.
(4) The phrase “states of mind regarding attachment” is currently a cypher, and often no more revealing than “internal working models”. For instance it remains unknown whether unresolved states of mind is a construct that offers any incremental validity over conventional measures of dissociation and posttraumatic stress.
(5) Relatedly, there especially appear to be breakdowns of communication when the concept of “trauma” is invoked in attachment discourses. There are usually some among the varied meanings of the term that both resonate and feel urgent for each person. This allows for good-natured and mutually convenient misunderstandings – especially between developmental researchers and psychotherapists.
(6) Researchers may wish to clarify the kind of “dysregulation” under discussion, rather than leaning on the connotations of the term. For instance, emotional flooding and constricted depression could both be considered dysregulated, but may well have different causes and implications (cf. Siegel, 1999, on the “Window of Tolerance”).

More generally, our conclusion here is that both advocates and critics of attachment theory have paid too little attention to differences in the ways that the theory has been adapted to the challenges and opportunities of its different domains of application. Tables 1–5 in the supplemental material seek to illustrate this point by displaying how the terms “attachment”, “security”, “internal working model”, “attachment-related trauma”, and “disorganisation” – though appearing to refer to a common object – in fact are given different meanings. We have discussed other cases elsewhere (e.g. Duschinsky & Foster, 2020, on “adaptation”). Different meanings referred to with a common term are liable to cause confusion, especially to those who are exposed to, users of, or contributors to more than one of the above discourses. For example in the case of “internal working model”, by which Bowlby meant expectations about the availability of the attachment figure, social psychologists mean elaborated and cognitive representations of the attachment figures and the self, and which Main et al. (1985) initially characterised as the construct measured by the AAI. This has led to a lack of specification as to what the AAI measures (it is not, as the name suggests, “adult attachment”). It has also contributed to communication barriers between developmental and social psychologists, for example in formulating hypotheses about when the AAI and ECR are expected to converge and diverge. In addition to causing confusion, unacknowledged differences in meaning also hinder the opportunity to make use of the knowledge and strengths of each domain, and direct attention away from the need to foster infrastructure to support coordination and mutual intelligibility across different domains. For example, such infrastructure might include forums to facilitate co-development of research agendas. The development of this infrastructure will be hindered if, for example, child “security” is assumed to be a goal, but actors take this to mean different things (see Table 2 for the varying meanings of attachment security across domains). For instance accurate and appropriate use of attachment theory, research and instruments – including concepts such as security – in the family courts has been obstructed by the difficulties in sustaining mutually enriching dialogue between researchers and court practitioners. The latter have had little ability to influence the direction of research, and there have been few professional rewards for researchers to address applied questions or refine and validate instruments for application in the court context (Garber, 2009).

In general, we advocate greater mutual intelligibility between domains, and hold that this may be facilitated by awareness of the technical use of terminology by academic researchers. At the same time we would urge that differences in perspectives and skills between domains are recognised and used. This would not only be the “transmission” of knowledge from high status knowledge to low status discourses. For instance: developmental attachment research could learn from popular attachment discourse, since the latter does not limit the concept of security to close relationships, and opens the question of what bidirectional links there may be between other sources of security (e.g. body confidence) and attachment relationships. This would return the field to questions that Ainsworth (2010, p. 49) felt had been neglected: “By focusing so closely on intimacies some attachment researchers have come to conceive of them as the only source of security – which is a pity”. Or again, attention to attachment in guidance to therapists is concerned with the specific effects of security, irreducible to the absence of attachment anxiety and avoidance; social psychological attachment research might benefit from attention to this literature if it proves willing to acknowledge evidence of limitations to the two-factor model.

The goals and adjustments of different domains of attachment discourse have contributed to their respective strengths. More focal awareness of these differences would, we feel, help reduce miscommunication and facilitate the coordination of different forms of knowledge: in collaborations between researchers and practitioners; in attempts by researchers to influence or draw from clinical and child welfare practice; in work to further articulate concepts and refine their scientific operationalisation; in clarifying assumptions when speaking to students and general audiences; and in efforts to draw from and integrate ideas and findings from developmental and social psychological attachment research.

## Figures and Tables

**Table 1:** Typification of differences in conceptualisations of “attachment”.

“Attachment”	
Popular Discourses	The child’s love for a parent, predominantly the mother; it is often utilised to signal moral expectations on the parent.
Developmental Science	The use of a caregiving figure as a safe haven (as well as potentially a secure base) signalling the history of the caregiving relationship.
Social Psychological Science	Close relationships with emotion regulatory functions, signalling the extent of anxiety or avoidance an individual experiences in these relationships.
Psychotherapy	Close relationships with emotion regulatory functions, signalling the extent of individuals’ difficulties with relational and self-understanding.
Psychiatric Diagnosis	The disposition to discriminate and seek a familiar caregiver when alarmed, signalling the existence of an attachment relationship as the basis for mental health.
Child Welfare Practice	The relationship quality between the child and their caregiver, signalling the child’s best interest.

**Table 2:** Typification of differences in conceptualisations of “security”.

“Security”	
Popular Discourses	A good and confident psychological state, and is presented as a desired state for everyone.
Developmental Science	The perceived availability of a safe haven in one’s attachment figure(s) (“felt security”).
Social Psychological Science	The absence of attachment anxiety and avoidance.
Psychotherapy	The mechanism of good mental health in the therapeutic relationship, and in a client’s other interactions.
Child Welfare Practice	A good parent-child relationship, indexing a child’s best interest.

**Table 3:** Typification of differences in conceptualisations of “internal working model”.

Internal Working Model	
Developmental Science	Used variously to mean: Expectations about the availability of attachment figures built up on the basis of repeated sequences of procedural interaction. Elaborated symbolic meanings and images built up by humans about attachment figures and their availability. A synonym for attachment representations, as used by Main in the 1980s (but subsequently abandoned).
Social Psychological Science	The elaborated symbolic and affective representations made by humans about attachment figures and their availability, and the value of the self to these attachment figures.
Psychotherapy	Elaborated conscious and unconscious symbolic meanings and images held by humans about attachment figures and their availability, and considered to be malleable through therapy.

**Table 4:** Typification of differences in conceptualisations of “attachment-related trauma”.

“Attachment-Related Trauma”	
Popular Discourses	Separations and other disruptions of the “natural” family.
Developmental Science	Negative impact of adverse events like loss and abuse on an individual’s current psychological state, evidenced by disoriented, incoherent discussion of these events
Social Psychological Science	An adverse event presumed to be a predisposing factor or cause of attachment anxiety and avoidance and/or an adverse event made psychologically disruptive by preexisting attachment insecurity.
Psychotherapy	Any experience from family life that is chronically disruptive of an individual’s internal and external regulatory capabilities. Includes abuse and neglect from caregivers or other trusted adults. Sometimes also referred to as “developmental trauma”.
Psychiatric Diagnosis	DSM/ICD definition of posttraumatic stress disorder.
Child Welfare Practice	Experiences that compromise the health and development of a child, and occurring in the context of the parent-child relationship.

**Table 5:** Typification of differences in conceptualisations of “disorganisation”.

“Disorganisation”	
Developmental Science	Used variously to mean: Conflict, confusion and/or apprehension shown by an infant towards their caregiver in the Strange Situation. A significant disruption of a behavioural system. It is this (invisible) disruption at the level of motivation, which is presumed to cause the visible conflicted, confused or apprehensive behaviour seen, for instance, in the Strange Situation. A category label for infant-caregiver dyads seen in the Strange Situation, where conflicted, confused and/or apprehensive behaviour is seen to a significant degree. The category label for controlling-punitive and controlling-caregiving behaviour in the Main and Cassidy 6-year reunion system. The behaviour was generally smoothly sequenced, goal-oriented, and often resulted in some form of caregiver availability – so it was not technically disorganised at a behavioural level. However, Main and Cassidy used the term “disorganised” to signal developmental continuities from infancy, and to high-light that controlling-punitive and controlling-caregiving behaviour likely arises in the context of disruption to the child-caregiver relationship and its usual hierarchies. The psychological process indicated by unresolved loss and trauma in the Adult Attachment Interview.
Social Psychological Science	The co-presence of attachment anxiety and avoidance and/or random chaotic behaviour.
Psychotherapy	A mechanism underpinning the contribution of emotion dysregulation to mental ill health.
Child Welfare Practice	A bad parent-child relationship, indexing a failure to align with a child’s best interest.
